# Building bridges to immunotherapy: a knowledge-mapping analysis of cervical cancer organoid research

**DOI:** 10.3389/fimmu.2026.1788725

**Published:** 2026-08-24

**Authors:** Yuanyuan Tian, Hui Chang, Jinjin Liu, Wenjun Ding, Jian Jin, Chi Qin, Shuoshi Zhao, Xin Zhao

**Affiliations:** 1 Department of Clinical Research and Translational Medicine, the Third Affiliated Hospital of Zhengzhou University, Zhengzhou, Henan, China; 2 Tianjian Laboratory of Advanced Biomedical Sciences, Institute of Advanced Biomedical Sciences, Zhengzhou University, Zhengzhou, Henan, China

**Keywords:** 3D culture, bibliometric analysis, cervical cancer organoids, human papillomavirus, precision medicine

## Abstract

Cervical cancer, a human papillomavirus (HPV) -driven malignancy with inherent viral antigenicity, serves as a key model in immuno-oncology. Yet, the variable efficacy of immune checkpoint inhibitors highlights a critical gap: the lack of preclinical models that accurately mirror the patient-specific tumor microenvironment (TME). Patient-derived organoids (PDOs) offer a transformative solution, preserving autologous tumor and immune components for personalized therapeutic testing. This study provides the first bibliometric analysis of this evolving field, mapping its knowledge structure from model development to immuno-oncological application. By analyzing 130 publications (1992-2025) from Web of Science, PubMed, and Scopus using VOSviewer and CiteSpace, we delineate a clear, three-stage evolution: from foundational HPV virology and culture techniques, through pivotal organoid methodology establishment (e.g., Burk RD, 2017), to the current translational phase. This progression is marked by citation bursts in TME modeling (e.g., Lohmussaar K, 2021) and the emergence of keywords like “drug screening” and “immunotherapy”. Research is dominated by the U.S., China, and European nations, published within an interdisciplinary core of oncology, immunology, and virology journals. Our analysis highlights the role of organoids as a crucial bridge linking basic virology to clinical immuno-oncology. However, standardization hurdles and suboptimal establishment rates for certain subtypes impede clinical translation. To realize the full potential of PDOs, future work must prioritize standardized immune-competent co-culture systems, integrate AI for high-content data analysis, and promote equitable global collaborations. Our analysis suggests the role of organoids as a crucial bridge linking basic virology to clinical immuno-oncology, highlighting that current advancements in tumor microenvironment modeling are establishing the necessary foundation for future immunotherapeutic applications.

## 1 Introduction

Cervical Cancer is the fourth most common malignant tumor among women worldwide. According to statistics from the World Health Organization (WHO), there were approximately 604,000 new cases and 342,000 deaths in 2020, among which about 90% occurred in low - and middle-income countries (1). Although the promotion of HPV vaccines and cervical cancer screening (such as TCT, HPV-DNA testing) have significantly reduced the incidence of early cervical cancer, the treatment of patients with advanced, metastatic or recurrent cervical cancer still faces severe challenges (2). The current standard treatment modalities include surgery, radiotherapy and chemotherapy, and targeted therapy (such as the anti-angiogenic drug bevacizumab), but the 5-year survival rate is still less than 20% in advanced patients (3). Key clinical challenges include: Tumor heterogeneity and drug resistance: Cervical cancer is highly heterogeneous at the molecular level. The interaction between oncogenic mechanisms driven by HPV infection (such as HPV16/18), such as oncoproteins E6/E7, and host genomic instability. This leads to significant differences in patients’ responses to chemotherapy (such as cisplatin) or radiotherapy (4). Lack of precise prediction models: Traditional 2D cell culture cannot simulate the complex structure of TME, while the mouse xenotransplantation model is costly, has a long cycle, and is difficult to reflect the human immune microenvironment (5–7).

This limitation stems from the fact that patient-derived xenograft (PDX) models rely on immunodeficient mouse hosts that inherently lack intact human immune systems. Thus, PDXs are unsuitable for investigating key components of the human TME (such as crosstalk between tumors and T cells or macrophages), nor can they assess the efficacy of immunotherapies that depend on a complete human immune background (8). In contrast, PDOs are established *in vitro* and can better preserve the native human TME of the original tumor. Notably, with advanced co-culture techniques, PDOs can maintain patient-specific cancer-associated fibroblasts (CAFs) and, more importantly, can be co-cultured with autologous immune cells isolated from the same patient (9, 10). This unique capability allows for the reconstitution of a patient-specific “immuno-oncology” platform, enabling the direct testing of immunotherapy responses and the study of mechanisms of immune evasion in a controlled yet human-relevant system. Therefore, beyond cost and speed, the superior ability of organoids to retain and recapitulate the human-specific TME represents their most significant advantage over PDX models for translational research, particularly in the era of cancer immunotherapy.

Accumulating molecular evidence has revealed the genetic variation characteristics of HPV16 E6/E7 oncoproteins among different populations, providing fundamental molecular basis for constructing patient-specific cervical cancer organoid models and interpreting heterogeneous immunotherapy responses. Relevant genomic analysis of HPV16 variants has deepened our understanding of viral oncogenesis and host–virus interaction, laying a molecular foundation for precision tumor modeling (11). Meanwhile, the rapid advancement of precision medicine and targeted therapeutic strategies has profoundly transformed contemporary cancer research, offering new theoretical support for the clinical translation of cervical cancer organoid-based drug screening and immunotherapy evaluation (12).

Individualized treatment demand: The response rate of immune checkpoint inhibitors (such as PD-1/PD-L1 inhibitors) in cervical cancer is only 10%-20%, and there is an urgent need for biological models that can predict treatment responses (13). In recent years, the rapid development of cervical cancer organoids has provided revolutionary tools for tumor biology research and precision medicine. Organoids are miniature organoid structures formed by culturing patient-derived tumor stem cells (CSCs) or tissue fragments in a 3D matrix, which can highly preserve the histopopathological characteristics, molecular heterogeneity and key components of the TME of the primary tumor (14). Hans Clevers et al. successfully established an HPV-positive cervical cancer organoid model for the first time, confirming that it can be passed on for a long time and maintain the expression of HPV E6/E7 oncogenes (15). Since then, several studies have optimized the culture system: Matrigel or collagen matrix supports organoid growth, while adding factors such as R-spondin 1 and EGF can enhance stem cell activity (15, 16). In addition to surgical specimens, puncture biopsy or organoids derived from circulating tumor cells (CTCs) offer research possibilities for advanced patients (17, 18). By knocking in the HPV gene through CRISPR technology, an isogenic model for etiological research can be constructed (19–21). Cervical cancer organoids have been widely used in: for example, drug screening: organoid drug sensitivity tests (such as cisplatin, palilizumab) are highly consistent with the clinical response of patients (22). Radiotherapy research: Organoids can simulate the differences in DNA damage repair after radiotherapy and reveal the mechanism of radioresistance (23). Immune microenvironment reconstruction: Co-culture of organoids with T cells or CAFs can evaluate the efficacy of immunotherapy (24). Despite the broad prospects, cervical cancer organoids still face challenges and limitations: such as differences in culture success rates: the organoid strain establishment rate for HPV-negative or poorly differentiated tumors is less than 30% (25). Lack of standardization: The culture process and drug sensitivity determination criteria have not been unified yet (26, 27).

Bibliometrics, as an important branch of scientometrics, provides an objective basis for research trend prediction and scientific research decision-making by quantitatively analyzing the production patterns, cooperation networks and knowledge structures of academic literature (28). In the field of biomedicine, bibliometrics has been widely applied in identifying research hotspots, evaluating academic influence and optimizing resource allocation (29). In recent years, the application of bibliometrics in the field of organoids has gradually increased. For example: Technology Evolution analysis: Wan et al. analyzed the emergent words in organoid research from 2000 to 2023 through CiteSpace and found that the research focus shifted from “intestinal organoids” to “tumor organoids” and “precision medicine” (30). Qu et al. utilized VOSviewer to reveal the core positions of the United States, China, and Europe in organoid research and pointed out the issue of insufficient cross-institutional collaboration (31). Xu et al. conducted a co-citation analysis of the literature on cancer organoids and identified “drug screening”, “tumor microenvironment”, and “organ-on-a-chip” as the three major clustering themes (32). However, most of the existing studies focus on the overall development of organoid technology, and the bibliometric analysis of the subfield of cervical cancer organoids is still blank.

The unique value of conducting bibliometric analysis on cervical cancer organoids. Firstly, through bibliometrics, this study can reveal research trends such as quantifying the annual number of published papers, core countries/institutions, and reflecting the field activity (33). Identify the knowledge base: Locate foundational literature through co-citation analysis (34). Predicting frontier trends: Emergent word detection (such as “immune organoids” and “AI-assisted drug screening”) can indicate the future direction (29). For instance, machine learning models are being developed to automate the analysis of organoid drug responses from high-throughput imaging, vastly improving the scalability and objectivity of screening assays (25). Optimize the research layout: Through cooperative network analysis, promote the formation of interdisciplinary teams (such as the cooperation gap between clinicians and bioengineers).

As the first comprehensive bibliometric mapping focusing on cervical cancer organoid research, this study systematically delineates the field’s rapid developmental trajectory and intellectual framework. Our results illustrate a distinct evolutionary path: from foundational model construction to in-depth TME research and subsequent clinical translational applications. Key challenges, such as the standardization of culture protocols and low success rates for certain subtypes, were identified as major barriers to clinical adoption. Looking forward, future efforts should prioritize establishing robust co-culture systems to better mimic the TME, integrating multi-omics data with AI-driven drug screening platforms, and fostering global collaborations to accelerate the development of personalized therapies. This study provides a foundational roadmap to guide these future endeavors.

## 2 Methods

### 2.1 Data acquisition

To construct a comprehensive knowledge base for this analysis, we sourced literature from three principal databases: Web of Science Core Collection (WOSCC), PubMed, and Scopus. Their selection was based on proven coverage of high-quality journals and broad disciplinary scope relevant to biomedical research. Specifically, WOSCC provides complete standardized citation metadata required for co-citation analysis, citation burst detection and clustering visualization via CiteSpace and VOSviewer. PubMed integrates authoritative clinical and immunology studies of cervical cancer with standardized Medical Subject Headings (MeSH) subject terms, fully capturing preclinical research focusing on organoid-based immunotherapy. Scopus complements a large volume of mid-tier biomedical journals and recent translational articles that are partially underrepresented in WOSCC. Databases including Google Scholar were excluded because they lack unified export citation formats and contain unfiltered grey literature, which cannot support standardized quantitative bibliometric clustering analysis.

The exact search string utilized was: (“uter cerv cancer*” OR “uter* cerv* carcinoma*” OR “uter* cerv* neoplas*” OR “cervical cancer*” OR “cervix cancer*” OR “cerv* cancer*” OR “cerv* carcinoma*”) AND (“organoid*” OR “tumoroid*” OR “tumor organoid*” OR “tumour organoid*” OR “patient-derived organoid*” OR “PDO” OR “stem cell-derived organoid*” OR “organ-on-a-chip” OR “organotypic culture”). The initial retrieval yielded a total of 286 raw records across the databases (PubMed: N = 71; Scopus: N = 143; WOSCC: N = 72).

**Figure 1:**
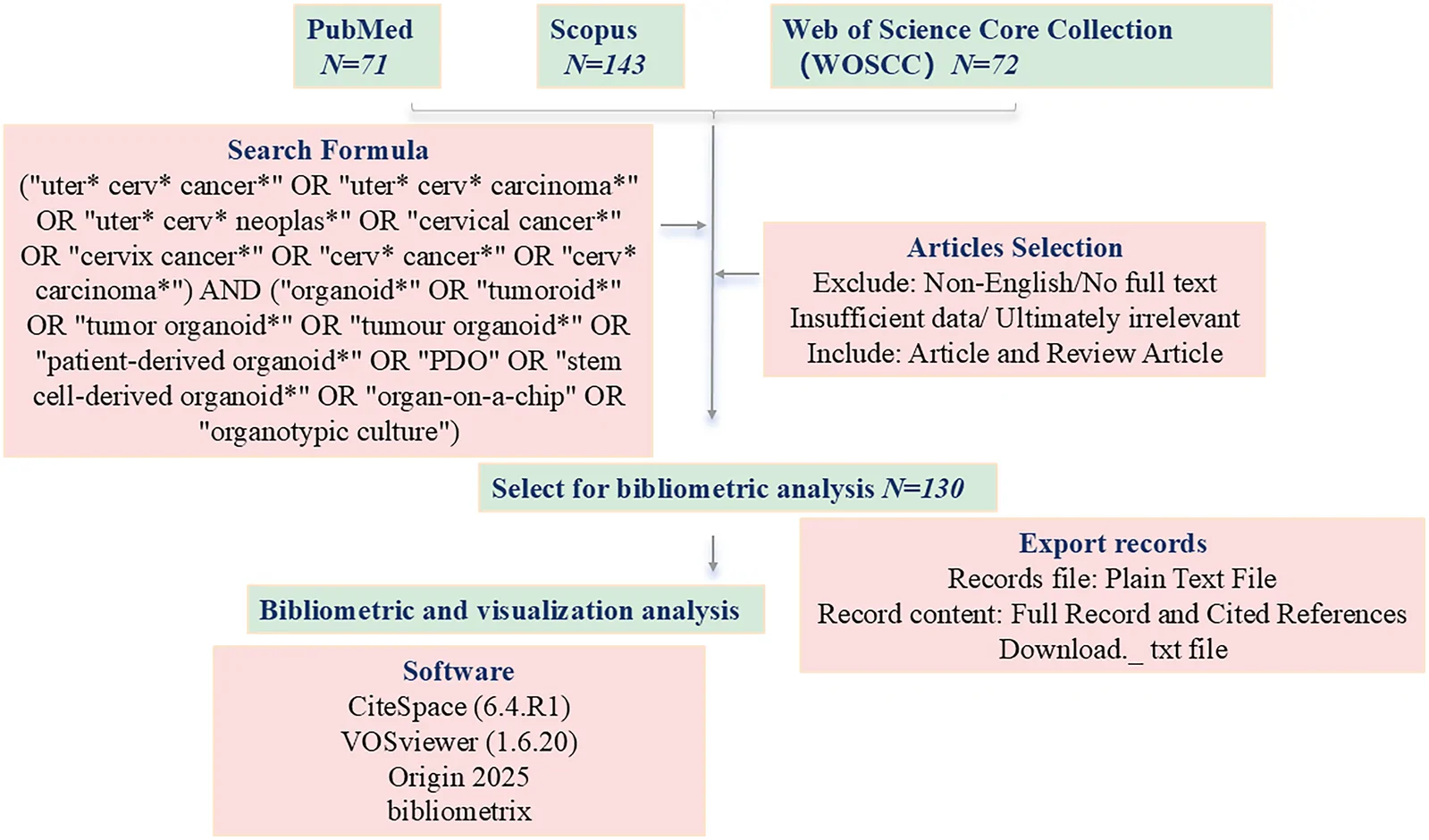
Workflow of bibliometric analysis for cervical cancer organoid research.

The deduplication workflow and subsequent screening were strictly executed as illustrated in **Figure 1**. After identifying and removing duplicate records computationally and manually, rigorous exclusion criteria were applied. We excluded: (1) non-English publications and articles lacking full text; (2) non-research document types (e.g., meeting abstracts, preprints, editorials, and conference proceedings); and (3) studies fundamentally irrelevant to organoid modeling. After removing duplicates and ineligible entries, 130 publications were retained for final analysis (**Figure 1**). To mitigate bias from daily database updates, all searches were executed on a single date: January 1, 2026. The final corpus, comprising full citation records and references exported in plain text format, was consolidated into a csv. file to facilitate downstream bibliometric processing.

### 2.2 Data analysis

Bibliometric analysis is a statistical tool used for quantitative analysis of a large number of existing literatures (35). It achieves data aggregation by extracting and analyzing information related to publications such as titles, keywords, authors, sources, and abstracts. In this study, we adopted two bibliometric software, VOSviewer and CiteSpace, to analyze and visualize scientific mapping (36, 37). Bibliometric research not only includes evaluation indicators but also involves scientific mapping techniques. The evaluation indicators mainly focus on the number of publications, citation frequency, identification of highly cited papers, as well as the analysis of outstanding researchers, countries and academic journals (38). Scientific cartography, on the other hand, focuses on analytical methods such as co-author identity, citation patterns, co-citation analysis, bibliographic coupling, co-occurrence maps of keywords, and data clustering (39).

In this study, the publication count for countries/regions and institutions was based on the “full counting” method. This means that each publication is assigned to all countries/regions and institutions listed in its affiliation fields. Consequently, a co-authored paper involving multiple countries will contribute to the publication count of each participating country. While we acknowledge that this computational approach inherently leads to a statistical inflation of the absolute total sum (i.e., the sum of country/institution counts significantly exceeds the actual core dataset of 130 unique papers), it was purposefully selected. While this approach may lead to a total sum of publications that exceeds the actual number of papers, it accurately reflects the breadth of participation and contribution of each entity, ensuring that no contributing node in global collaborations is masked. The subsequent co-authorship network analysis complements this by visualizing the links between these entities.

### 2.3 Software parameter

This study conducted a joint analysis using VOSviewer and CiteSpace software (35, 40). The selected publication is exported in Refworks format and saved as the “download_txt” file. Each record contains information such as the author’s name, affiliation, publication date, title, abstract and keywords. The annual frequency statistics of the included publications were conducted using NoteExpress 3.6.0 software, and the histogram of the literature volume was plotted using Origin 2025. Import the “download_txt” file into VOSviewer 1.6.20 and CiteSpace 6.4.R1, and use VOSviewer 1.6.20 for co-occurrence analysis of authors, institutions and keywords. And the Origin 2025 was used to draw Sanji maps. Meanwhile, CiteSpace 6.4.R1 was utilized for keyword clustering, emergent analysis and timeline analysis. The parameter Settings are as follows: For keyword co-occurrence analysis, we set the minimum occurrence threshold of keywords to more than 5, and finally retained 52 qualified keywords for subsequent network mapping. In CiteSpace analysis, the time span was defined from 1992 to 2025, with one year per time slice. The node selection adopted the g-index strategy with a scaling factor of k=25, and the auxiliary filtering parameters were set as LRF = 3.0, L/N=10, LBY = 5, and e=1.0. The generated literature co-occurrence network contains 450 nodes and 1742 links, with a network density of 0.0172. To simplify redundant connections and highlight core knowledge associations, the Pathfinder algorithm was applied for network pruning, and the node label display ratio was set to 1.0% to balance information density and visual readability.

Clustering quality validation showed that the network Modularity Q index was 0.6653 and the Weighted Mean Silhouette coefficient was 0.8763, with a harmonic mean of 0.7563. A Modularity Q value greater than 0.3 indicates a statistically significant and well-structured clustering framework, while a Silhouette coefficient above 0.7 reflects high thematic homogeneity within each cluster. These metrics verify that our keyword clustering and knowledge mapping results are stable, reliable, and scientifically interpretable (41, 42).

### 2.4 Journal impact factor data

The Journal Impact Factors (JIF) cited in this study were sourced from the 2023 Journal Citation Reports (JCR) (Clarivate Analytics), released in June 2024. The JIFs are provided for informational context regarding the journal’s influence at the time of analysis and are accurate as of this source.

## 3 Results

### 3.1 Worldwide publication landscape

**Figure 2:**
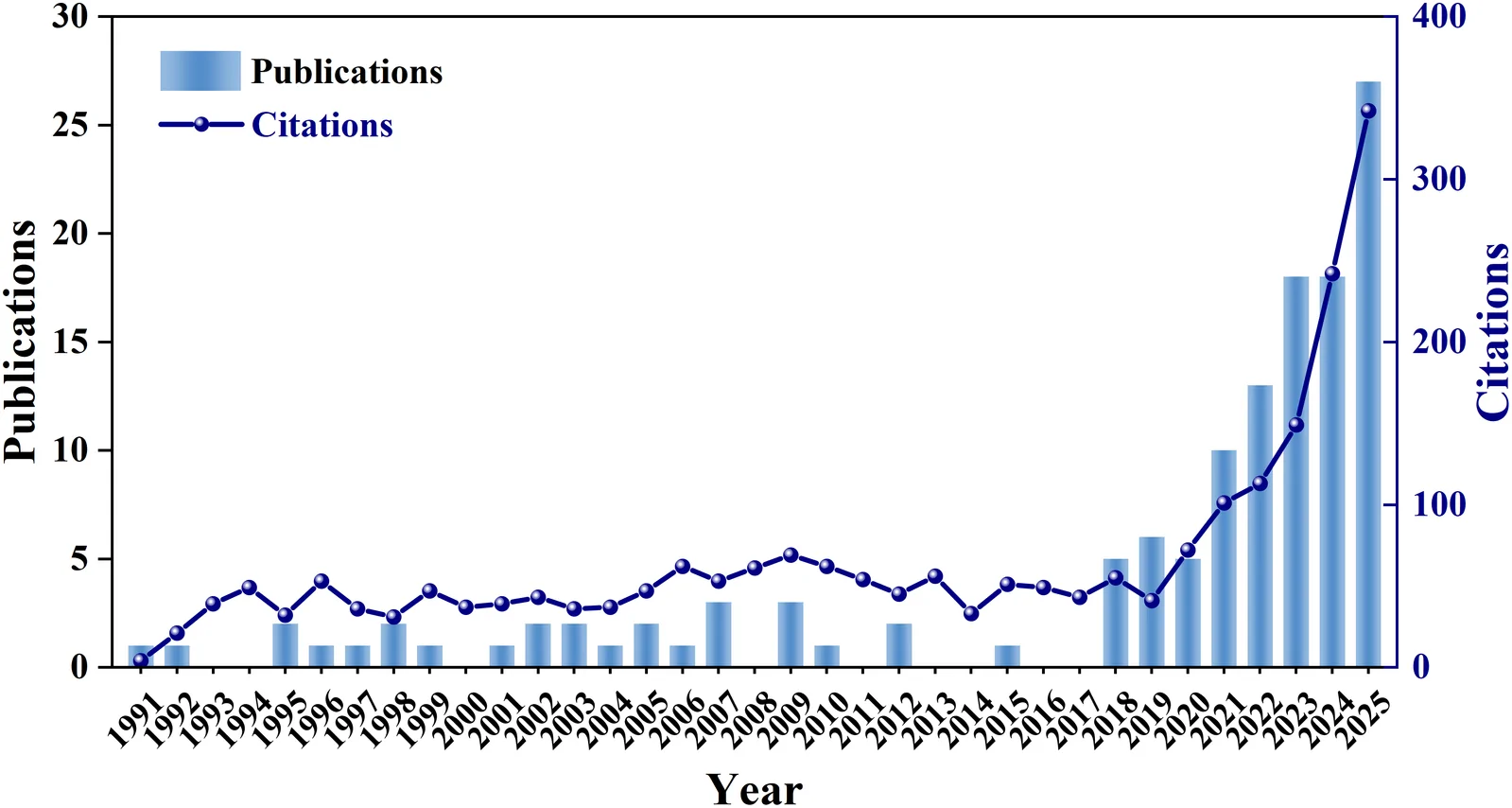
Annual trends in publications and citations of cervical cancer organoid research.

Research utilizing organotypic cultures for cervical cancer dates to the early 1990s. The broader field of organoid technology advanced significantly following the establishment of the first intestinal organoid in 2009. A pivotal milestone was reached in 2018, when researchers at the Hubrecht Institute (the team of Hans Clevers) first reported the successful generation of cervical cancer organoids (15). Since that foundational work, annual publication output in this niche has grown consistently, averaging between ten to twenty papers per year globally. As shown in **Figure 2**, both the annual number of publications and their corresponding citation counts have demonstrated a clear upward trajectory over time, with a peak in publication volume observed in 2025. This trend reflects a steadily increasing engagement with and focus on cervical cancer organoid models within the scientific community.

### 3.2 Countries, regions and institutions

**Figure 3:**
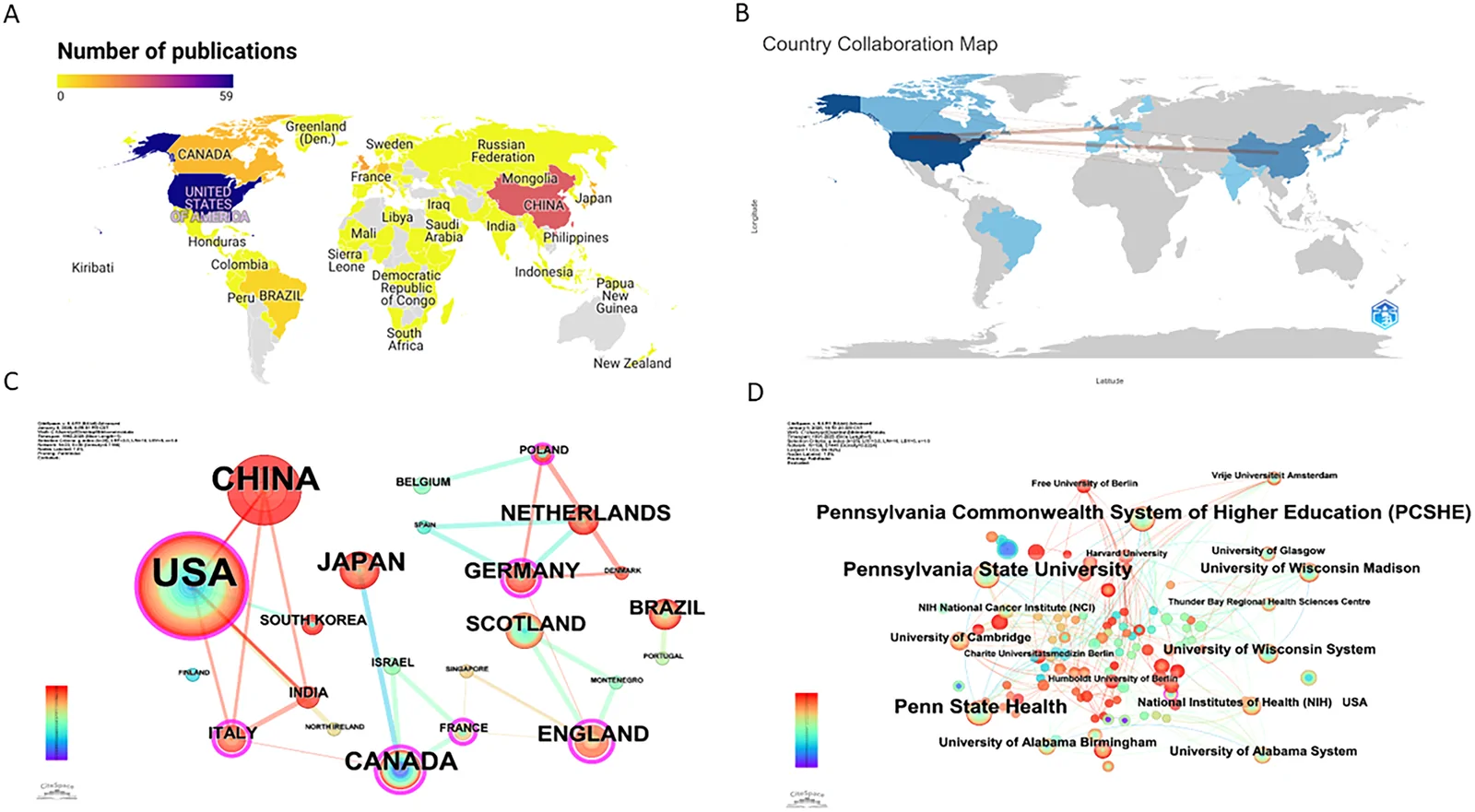
Visualization of cooperation among countries/regions and institutions. **(A)** The number of publications on cervical cancer organoids in different countries and regions. **(B)** Country Collaboration Map. The map illustrates the geographical distribution of collaborative research publications among countries and regions. **(C)** Country collaboration network visualized with CiteSpace. **(D)** Institutional collaboration network in academic research, visualized using CiteSpace.

Our analysis of publication origins reveals the global landscape of cervical cancer organoid research. A total of 23 countries and 199 institutions have contributed to the literature. Using a full counting method (where each country on a multi-national publication receives credit), the United States led in contribution frequency (n=59), followed by China (n=28), with Canada and Japan (n=11 each) also making substantial contributions. Significant output originated from European nations including Germany, the Netherlands, and England (n=8 each), as well as Scotland (n=7) and Brazil (n=6) in South America (**Figure 3A**). International collaboration is a hallmark of the field. The United States emerged as the most active collaborative hub, engaging with 11 distinct countries. Its strongest bilateral partnership was with China, followed by France, indicating prominent trans-Pacific and trans-Atlantic knowledge exchange (**Figure 3B**). This is visualized in the co-authorship network (**Figure 3C**), where the USA, China, and Germany form the largest nodes. Dense interlinks between Western Europe, North America, and East Asia underscore a robust trans-continental collaborative core. At the institutional level, this cooperation evolved from limited, early partnerships into a complex, cross-border network. Major hubs include the NIH-NCI, Harvard University, and Charité - Universitätsmedizin Berlin. Notably, dense trans-Atlantic ties among the University of Wisconsin-Madison, Pennsylvania State University, and the University of Cambridge highlight a powerful US-EU axis driving the field forward (**Figure 3D**).

### 3.3 Evaluation of co-authorship networks and core-author distributions

**Figure 4:**
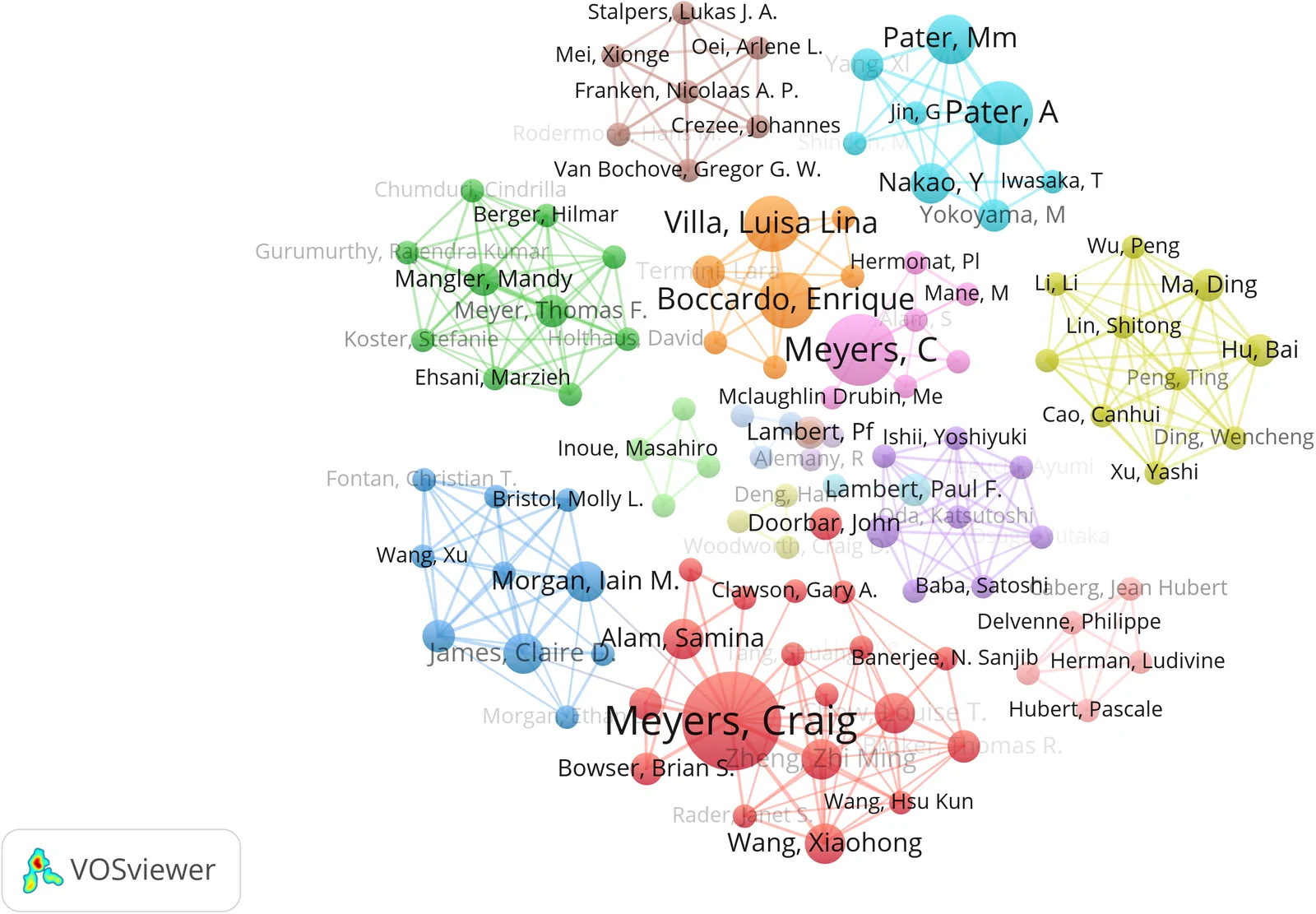
Author co-authorship network. (Node size corresponds to an author’s total connection strength, while link thickness represents the frequency of collaboration).

**Figure 4** maps the co-authorship network within the cervical cancer organoid literature (encompassing the core dataset of 130 publications from 1992-2025). The analysis identifies 109 authors, grouped into 20 distinct, color-coded collaborative communities. In this network visualization, the size of a node reflects the author’s total publication volume and connection strength; the thickness of the lines linking them indicates the frequency of their co-authorship; and the clustering colors denote distinct collaborative communities, often grouped by geography or specialized topics. The network reveals specialized geography-linked clusters: Cluster 1 (red), anchored by Craig Meyers and NIH-associated groups, forms a translational hub focused on viral vector applications and PD-L1 assay development in organoids. Cluster 2 (blue) comprises researchers from Charité-Humboldt, concentrating on HPV-16-specific 3D invasion models. Expertise is further segregated into thematic clusters, such as European radiochemoradiation specialists (Cluster 8, purple) and Asian research consortia (Cluster 4, green). A high density of connections between institutions like UW-Madison, Penn State, and Cambridge signifies a robust US-EU knowledge bridge. Overall, the network exhibits a core-periphery structure, with a tightly interconnected North American and European core driving foundational immunotherapy innovation, complemented by actively emerging East Asian clusters that are expanding the application of organoids in precision oncology.

### 3.4 Co-cited journals

**Figure 5:**
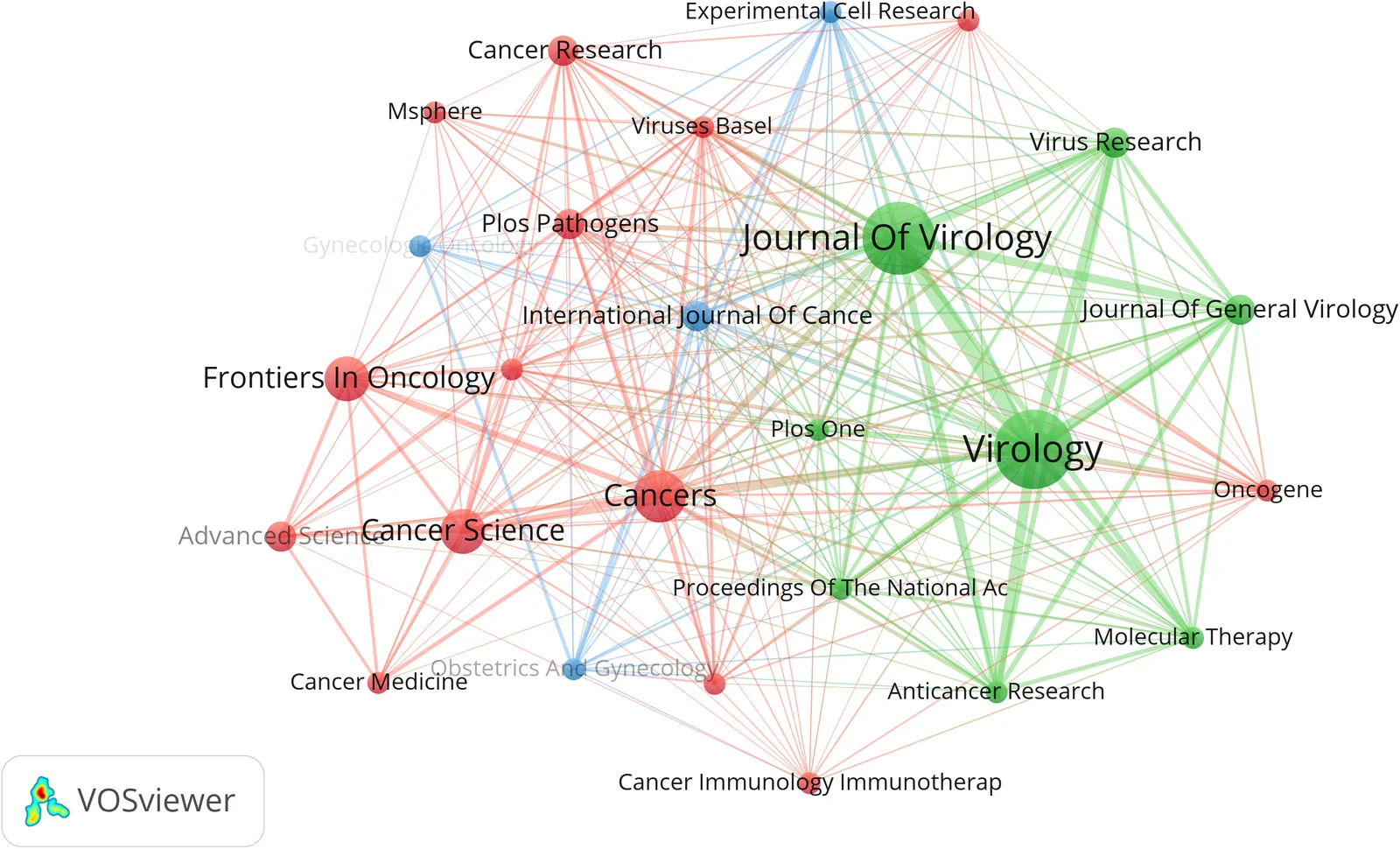
Network of journals based on co-citation. Node size indicating citation frequency, and links representing co-citation. Clusters may reflect interdisciplinary fields.

The journal co-occurrence network reveals a coherent, interdisciplinary knowledge structure supporting cervical cancer organoid research, marked by distinct bridges to immunology (**Figure 5**). In this co-citation map, node size represents the total citation frequency of the journal within our dataset, while the distance and links between nodes illustrate how often articles from these journals are cited together (co-citation strength). A dense core of high-impact experimental oncology journals anchors the network, with Cancer Research (IF: 16.6; Q1) acting as its principal hub. This core displays strong linkages to other major cancer journals, such as International Journal of Cancer (weight=281) and Frontiers in Oncology (weight=129) and is reinforced by titles like Oncogene and Cancer Science.

Critically, this oncological core is robustly connected to specialized immunology literature. Cancer Immunology, Immunotherapy (IF: 5.1; Q1) functions as a key bridge, with measurable co-citation weights to several core journals (e.g., weight=18 with Frontiers in Oncology), visually mapping the “bridge” to immunotherapy. The network further integrates essential supporting fields: virology journals (PLoS Pathogens, Virus Research) underscore the foundational HPV focus; premier multidisciplinary platforms (PNAS, Advanced Science) indicate broad validation; and clinical journals (Obstetrics and Gynecology) highlight translational relevance. Composed predominantly of Q1 and Q2 journals, this architecture indicates a mature, high-quality research front where knowledge actively flows from model development toward immunotherapeutic strategy evaluation.

### 3.5 Citation analysis

**Figure 6:**
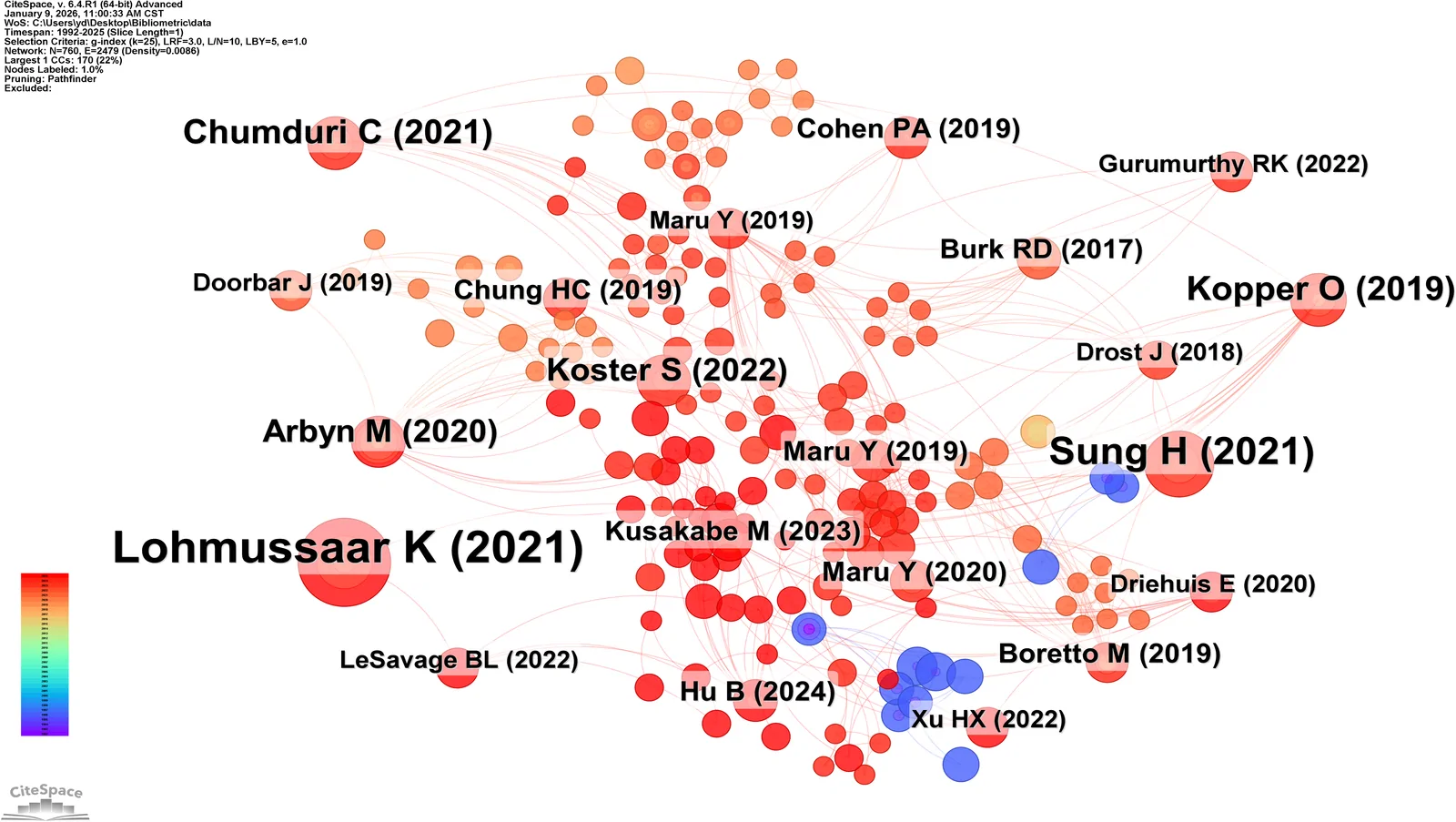
Document co-citation network. (Node size denotes the citation frequency of the reference, and links represent co-citation strength. This visualizes the core intellectual foundations of the field).

The document co-citation network (**Figure 6**) delineates the core intellectual foundations of cervical cancer organoid research. The network is anchored by seminal methodological papers on organoid generation, with the work of Lohmussaar K et al. (2021, Cell Stem Cell; frequency=20) serving as the most influential node. This methodological core, including Kopper O et al. (2019) and Boretto M et al. (2019), is robustly connected to foundational knowledge in HPV virology, exemplified by Doorbar J et al. (2019) and Burk RD et al. (2017), and to key clinical epidemiology studies like Arbyn M et al. (2020). This structure largely suggests that the field is built upon a triad of model development, viral etiology, and clinical understanding. Critically, pivotal nodes such as Chumduri C et al. (2021) not only contribute to organoid methodology but also explicitly investigate immune and host-pathogen interactions, thereby forming a direct conceptual bridge toward immunotherapy. The presence of recent, high-impact studies on tumor microenvironments (e.g., LeSavage BL et al., 2022) further indicates the field’s active evolution toward immune-oncology applications. Thus, the co-citation map visually traces the knowledge pathway from establishing basic models to leveraging them for immunotherapeutic discovery.

**Figure 7:**
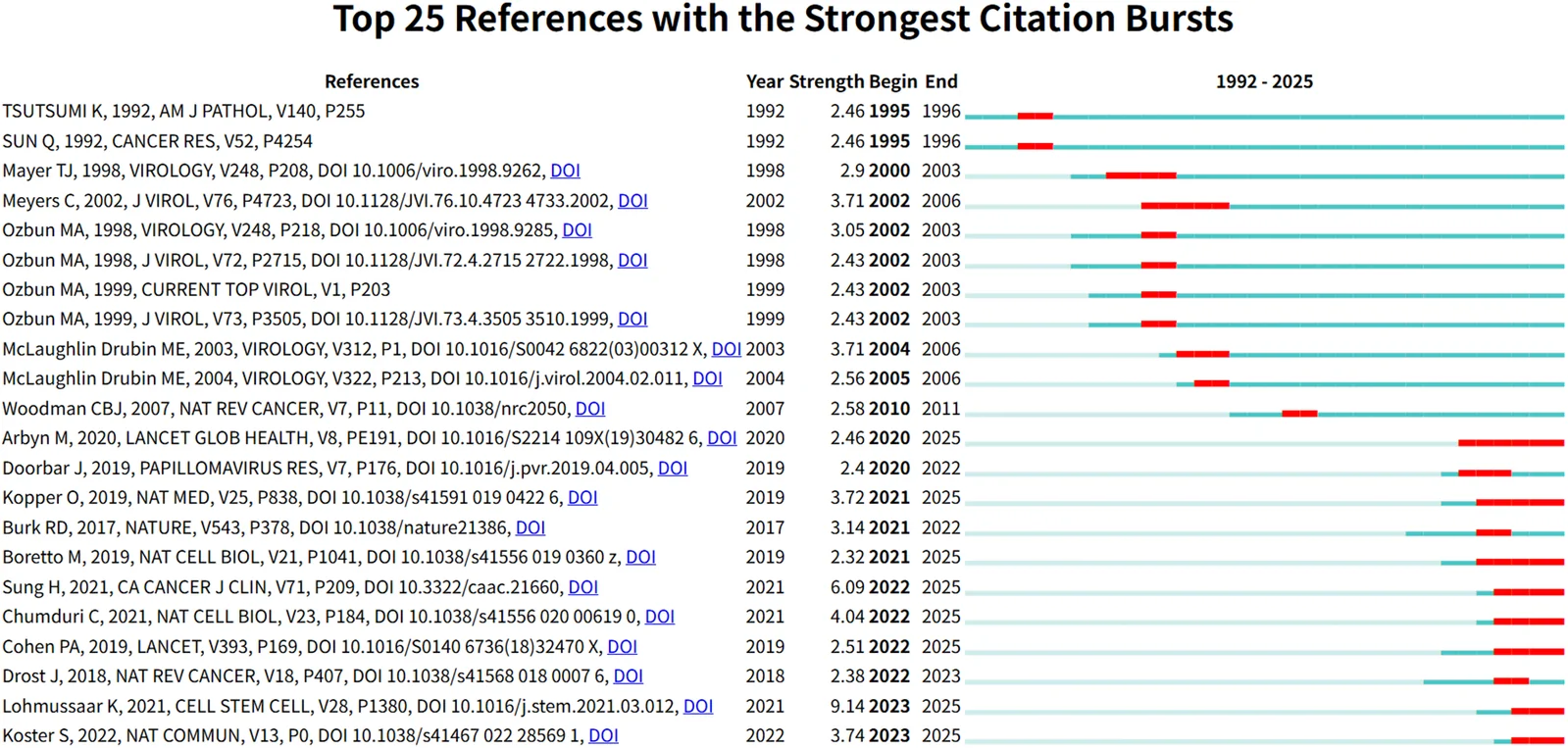
Top 25 references with the strongest citation bursts. The red segments on the timeline indicate the period during which a reference experienced a burst of citations, with the specific start and end years labeled. The strength of each burst is indicated by the value following the reference.

The analysis of citation bursts (**Figure 7**) reveals the temporal evolution of research fronts and the sharp rise of the organoid paradigm within cervical cancer research. The earliest bursts in the 1990s (e.g., Tsutsumi K et al., 1992) pertain to early histopathological and virological studies, establishing the foundational link between HPV and cervical lesions. A pronounced cluster of bursts between 2002-2006, dominated by work from Ozbun MA et al. and McLaughlin Drubin ME et al., represents a key phase of intense research into *in vitro* HPV life cycle modeling and raft culture systems, which were critical technological precursors to modern 3D organoids. The burst for Woodman CBJ et al. (2007), a seminal review, marks the consolidation of HPV carcinogenesis knowledge.

The most significant and recent bursts unequivocally signal the field’s current trajectory. Beginning around 2020-2021, a powerful new cluster emerges with exceptionally high burst strength. This includes landmark organoid methodology papers: Lohmussaar K et al. (2021, Cell Stem Cell) exhibits the highest burst strength (9.14), followed by Kopper O et al. (2019) and Boretto M et al. (2019), highlighting the explosive adoption of these models. Critically, the concurrent bursts of Chumduri C et al. (2021), which explores host-pathogen-immune interactions in organoids, and key clinical studies (1, Arbyn M et al., 2020, 13), suggest that the field’s rapid growth is not confined to basic model development. Instead, it is integrating high-impact clinical epidemiology and, most importantly, research that uses organoids to dissect the tumor microenvironment-a prerequisite for building bridges to immunotherapy. This pattern of bursts illustrates a clear chronological shift from foundational virology through enabling 3D culture technologies, culminating in the present explosive phase where organoids are actively deployed to address translational questions, directly paving the way for immunotherapeutic discovery.

### 3.6 Keywords analysis

**Figure 8:**
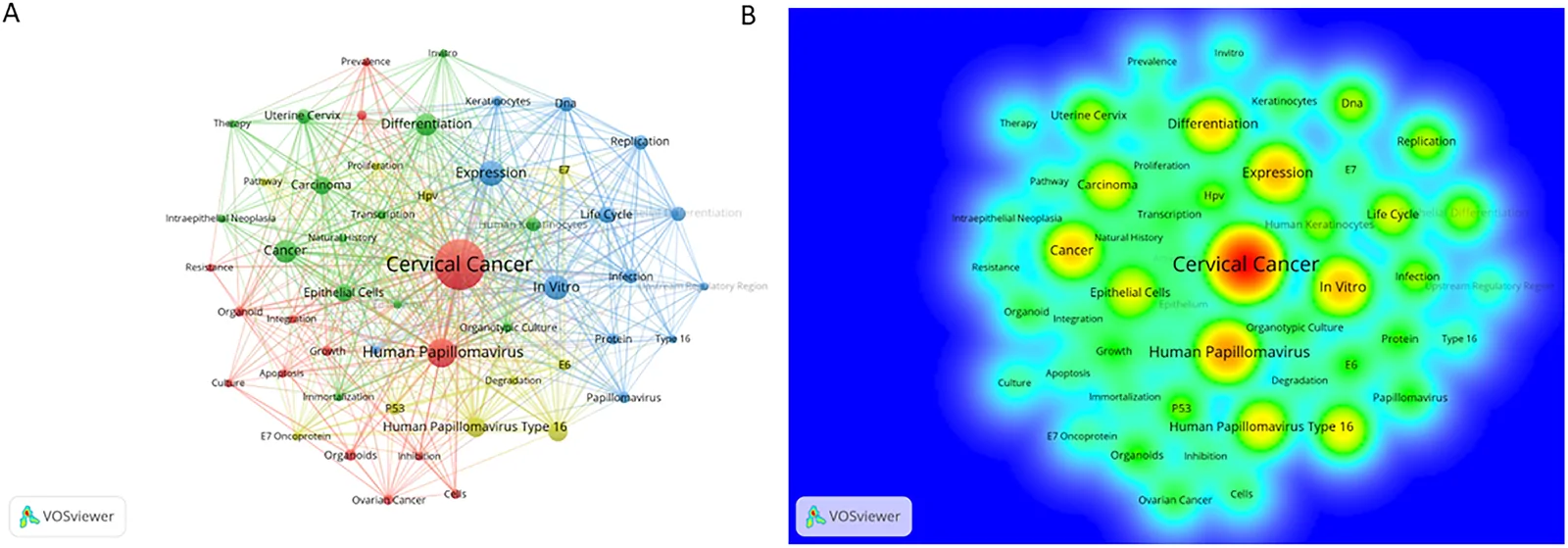
Analysis of keywords visualization. **(A)** The visual network diagram of keyword co-occurrence analysis. **(B)** Analysis of the keyword cloud image.

The keyword co-occurrence and clustering analysis (**Figures 8A, B**) delineates the core thematic structure and hot topics within cervical cancer organoid research, revealing both its deep roots in virology and its nascent, evolving focus toward therapeutic applications. In these visualizations, the size of a keyword node illustrates its absolute frequency of appearance, while link thickness reveals how frequently terms occur together in the same publication, with proximity indicating strong thematic relationships. The network is dominated by a high-frequency core cluster centered around “Cervical Cancer” (64 occurrences) and “Human Papillomavirus” (29 occurrences), signifying the foundational etiological anchor of the field. Tightly linked to this core are key virological and molecular biology terms, such as “HPV Type 16”, “E6”, “E7” “Degradation”, “p53”, “Replication”, and “Life Cycle”, which form a dense sub-cluster focused on the fundamental mechanisms of HPV-driven oncogenesis and viral-host interactions. Another prominent historical cluster revolves around “*in vitro*” and differentiation models, including “*In Vitro*”, “Keratinocytes”, “Epithelial Cells”, “Differentiation”, and “Organotypic Culture”. These keywords represent the crucial methodological evolution from traditional 2D cell culture and raft systems that preceded modern 3D organoids.

Critically, the keyword “Organoids” (8 occurrences) and “Organoid” (7 occurrences) emerge as distinct, recent nodes with high average publication years, highlighting this as the current, rapidly evolving methodological frontier. While still quantitatively smaller than the foundational clusters, their connections are highly informative. They are directly linked to the core HPV biology cluster, indicating that current organoid research is actively used to model these fundamental virological processes. More importantly, the analysis reveals several emerging bridges. The keyword “Therapy” appears in the network, and terms like “Resistance” and “Ovarian Cancer” (often studied alongside cervical cancer in organoid platforms for drug testing) indicate a growing translational focus. Although direct keywords like “immunotherapy” are not yet statistically dominant, indicating that the field is still in a critical transitional phase heavily focused on establishing robust biomimetic models; these platforms serve as essential precursor steps for building the specific bridge to immunotherapy. The presence of “Pathway”, “Activation”, and “Inhibition” within the network suggests the beginning of mechanistic, target-oriented research. Although explicit keywords like “immunotherapy” are not yet statistically dominant, indicating that the field is still in a critical transitional phase heavily focused on establishing robust biomimetic models-these platforms serve as essential precursor steps for building the specific bridge to immunotherapy. This apparent inconsistency between static keyword frequency and dynamic frontier signals arises from the developmental stage of the field: immunocompetent organoid and immunotherapy research only boomed after 2020, so cumulative absolute occurrences remain low. However, timeline mapping and citation burst metrics capture the rapid recent growth of this theme, qualifying it as an emerging hotspot rather than a mature, high-frequency core theme.

### 3.7 Analysis of hot spots

**Figure 9:**
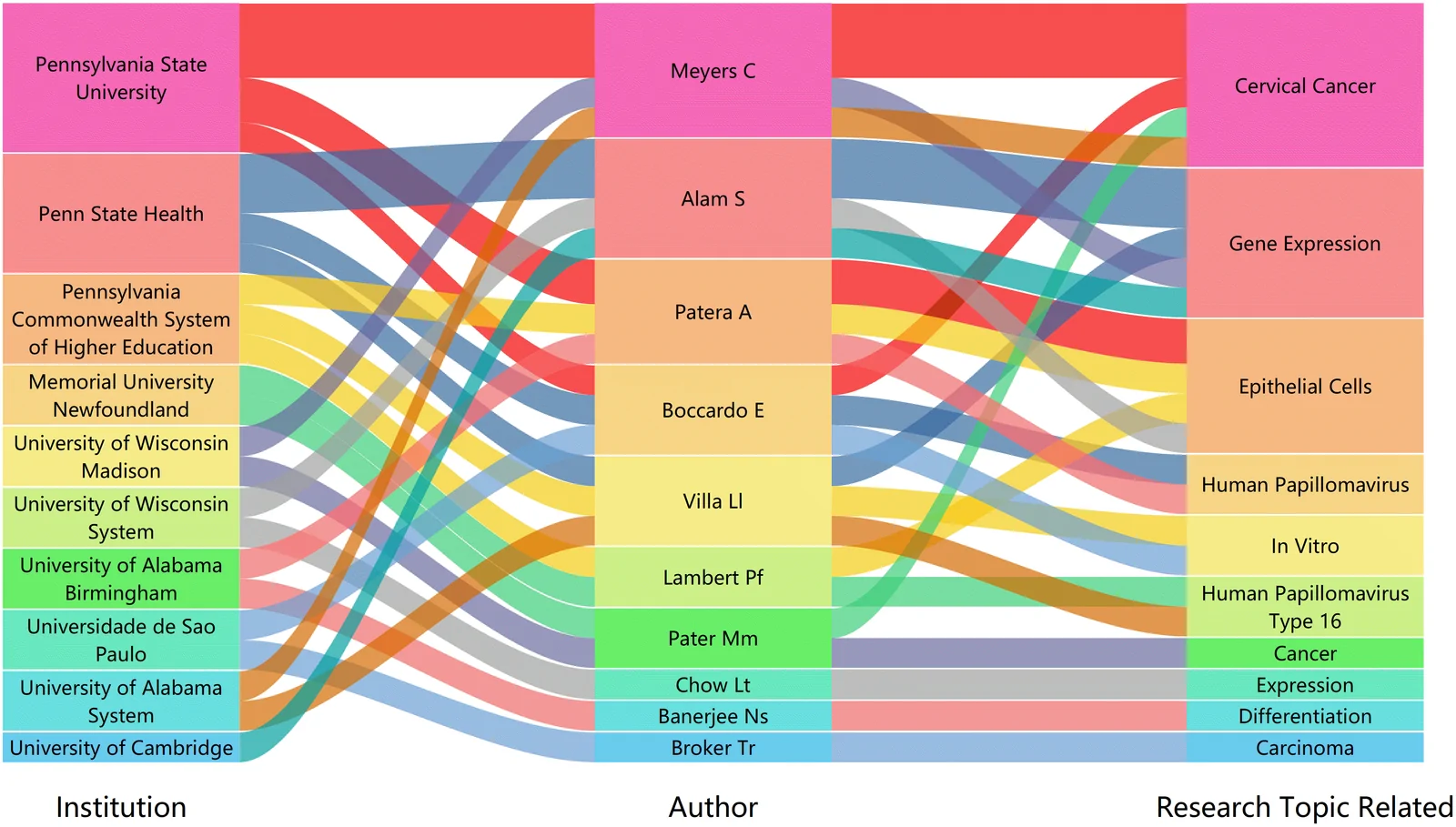
Sankey analysis of cervical cancer organoid research, linking institutions, author and research topic.

The Sankey plot (**Figure 9**) generated by the bibliometrix package of the R language was used to analyze the flow of contributions from major research institutions to prominent authors and their associated key research topics within the cervical cancer organoid knowledge domain, highlighting established centers of expertise. The analysis identifies several key institutional hubs that have historically anchored this field. “Pennsylvania State University (and its affiliated health system)” emerges as a significant contributor, linked to author “Meyers C”, whose work is foundational to modeling cervical cancer biology. Similarly, institutions like the “University of Wisconsin-Madison” and the “University of Alabama System” are connected to authors focused on core methodological and biological aspects, such as “*in vitro*” models, gene expression, and carcinogenesis. The international contribution is exemplified by “Universidade de Sao Paulo”, associated with author “Chow Lt”, who has contributed extensively to the understanding of HPV biology and epithelial differentiation-fundamental pillars for organoid culture development.

The mapped research topics predominantly cluster around the foundational elements of the field: “Cervical Cancer”, “Human Papillomavirus”, “*In Vitro*”, “Expression”, and “Differentiation”. This pattern confirms that the knowledge base for cervical cancer organoids is deeply rooted in institutions and authors with long-standing expertise in virology, epithelial cell biology, and traditional experimental models. While the diagram effectively captures these historical and foundational intellectual currents, it reflects the data used for the overall bibliometric mapping, which encompasses the field’s evolution. Therefore, the most recently emerging contributions from institutions pioneering patient-derived organoid models and their application in immunotherapy may not yet be as densely represented in this specific historical flow visualization. Nevertheless, the diagram successfully delineates the established academic lineages and core research streams that have provided the essential scientific groundwork upon which the current, more translational organoid and immunotherapy research is being built.

**Figure 10:**
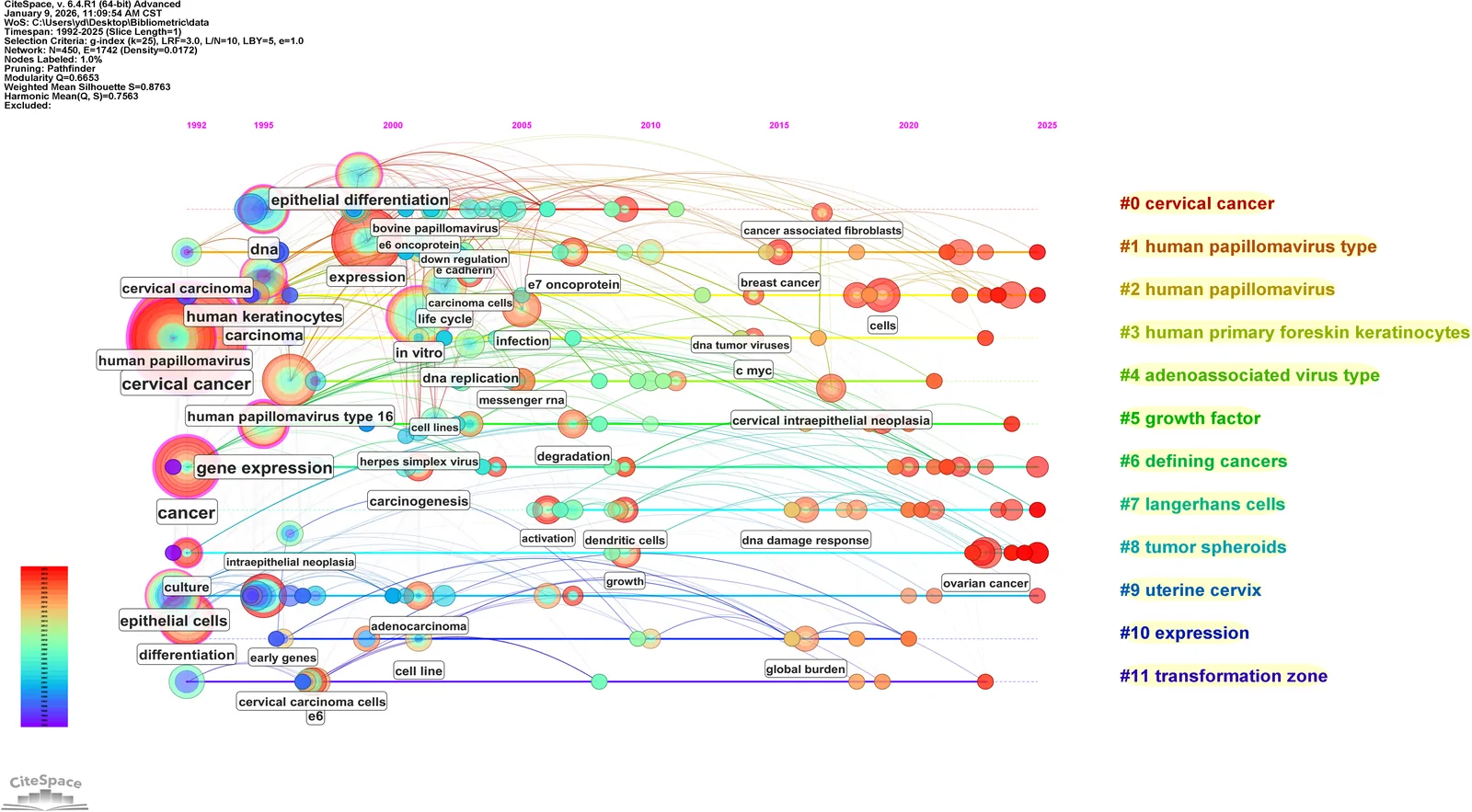
Citation relationships and logical grouping of themes related to cervical cancer organoid.

**Figure 10** delineates the keyword-timeline landscape of cervical-cancer-organoid from 1992-2025. “Cervical cancer” and “human papillomavirus” (centrality 0.38 and 0.12, respectively) anchor the earliest stratum, establishing the virological foundation of the field. A mid-stage cluster (1999-2007) pivots toward functional assays-”*in vitro*”, “epithelial differentiation”, “life cycle”-signaling the transition from descriptive studies to mechanistic culture systems. After 2015, neologisms such as “tumor organoids”, “3D culture”, “drug screening” and “precision medicine” emerge and persist, coinciding with the plateau of “cervical intraepithelial neoplasia” and “DNA damage response”; this lexical shift documents the community’s strategic move from monolayer models to immunocompetent organoid platforms for evaluating checkpoint inhibitors and combination therapies. Notably, “resistance” and “cisplatin” appear only after 2022, indicating that acquired therapeutic tolerance is now framed within a 3D tumor-microenvironment context. Overall, the timeline reveals a knowledge bridge: early HPV-centric oncogenesis vocabulary has been repurposed to serve an immunotherapy-oriented, organoid-based experimental repertoire, underscoring the field’s evolution toward translational precision oncology.

## 4 Discussion

### 4.1 Research highlights and tendencies

Notably, all biological, immunological and clinical mechanistic interpretations presented in this subsection are derived from independent original experimental studies cited throughout this manuscript. Bibliometric analysis only quantifies thematic trends and knowledge linkages within published literature; it cannot directly validate biological causality or clinical therapeutic efficacy. All mechanistic deductions below serve as auxiliary interpretations based on existing functional research rather than direct conclusions yielded by our bibliometric statistics.

Our bibliometric analysis delineates a clear and evolving intellectual landscape for cervical cancer organoid research, moving from foundational model establishment toward sophisticated clinical integration. The findings from keyword evolution (**Figure 10**), citation bursts (**Figure 7**), and clustering analysis (**Figure 8**) collectively reveal three predominant trends.

First, the field has undergone a clear thematic progression. Early research, represented by keywords such as “*in vitro*”, “keratinocytes”, and “differentiation” in the timeline analysis and high-citation works from the 1990s-2000s (e.g., Ozbun MA, 1998), was predominantly focused on overcoming technical hurdles in establishing 3D culture systems. Subsequently, the emphasis shifted decisively toward mechanistic exploration, particularly the role of “HPV” and its oncoproteins within these models. This is strongly supported by the citation burst of Burk RD’s (2017) seminal work and the tight keyword cluster linking “HPV”, “E7”, “p53”, and “degradation”. Most recently, the field has entered a translational phase, with emerging foci on “drug screening”, “immunotherapy”, and “precision medicine”, as evidenced by recent high-burst publications such as those by Sung H (1) and Koster S (2022).

Second, our co-occurrence and cluster analysis (**Figures 8A, B**) identify the simulation of the TME as a central and current research hotspot. The strong associations between keywords and the convergence of journals from immunology, virology, and interdisciplinary sciences underscore a concerted effort to develop more physiologically relevant co-culture models. Finally, the citation burst analysis and institutional collaboration mapping signal the rise of high-throughput applications and precision medicine, with organoids being rapidly adopted as platforms for large-scale compound testing and personalized therapeutic prediction. While our keyword analysis confirms that fundamental virological components (such as HPV Type 16, E6, and E7) remain central to the field, a critical biological paradox drives the recent shift toward TME modeling. Given that HPV-driven tumors express highly immunogenic viral oncoproteins (E6/E7), they are inherently antigenic. However, as highlighted by the variable clinical responses to immune checkpoint inhibitors (ICIs), many patients still fail immunotherapy. This clinical reality is prompting a surge in organoid research to uncover why. The literature suggests that prolonged viral infection induces a profound state of immune exhaustion, establishing a highly suppressive TME enriched with regulatory T cells (Tregs) and myeloid-derived suppressor cells (MDSCs). Checkpoint blockade alone is often insufficient to reverse this terminal exhaustion. Therefore, the bibliometric transition toward immunocompetent PDOs reflects the field’s urgent need to model these specific immunosuppressive networks *in vitro* and identify novel combination therapies that can sensitize “cold” or exhausted HPV-driven tumors.

The bibliometric rise of “drug screening” as a central node brings another critical structural shift into focus. High-throughput organoid screening inherently generates multi-dimensional data. While not yet forming an independent, massive keyword cluster, emerging literature intersecting with our network suggests a nascent reliance on Artificial Intelligence (AI) and machine learning. Rather than broad, speculative applications, recent linked studies focus densely on AI-assisted analysis of high-content imaging (HCI) to quantify dynamic spatial metrics (T cell infiltration), and integrating multi-omics data from PDOs to build models for drug sensitivity. Thus, the literature trajectory suggests AI is poised to act as the essential catalyst converting qualitative 3D cultures into the quantitative decision-making platforms envisioned by the “precision medicine” keyword burst.

Furthermore, as our citation analysis confirms the field’s enthusiastic embrace of TME modeling, a critical bibliometric pattern emerges regarding immunocompetent systems. Although “immunotherapy” is a rising keyword, highly cited critical reviews within our dataset highlight severe technical bottlenecks, particularly the rapid phenotypic drift of tumor-infiltrating lymphocytes (TILs) and the inability to preserve macrophage polarization ex vivo without artificial supplementation. Consequently, the recent peripheral emergence of terms like “organ-on-a-chip” in our systematic search (**Figure 1**) reflects a necessary methodological pivot. Achieving true clinical surrogacy will require the field to move beyond static co-cultures toward microfluidic systems that can sustain phenotypically stable immune components.

Ultimately, a critical question dictates the future intellectual structure of this field: Do PDO responses actually correlate with patient clinical outcomes? Recent highly cited translational studies identified in our analysis increasingly validate this link, demonstrating strong predictive values for standard chemoradiotherapy. However, regarding immunotherapy, direct correlation data remains in its infancy in the published networks.

**Figure 11:**
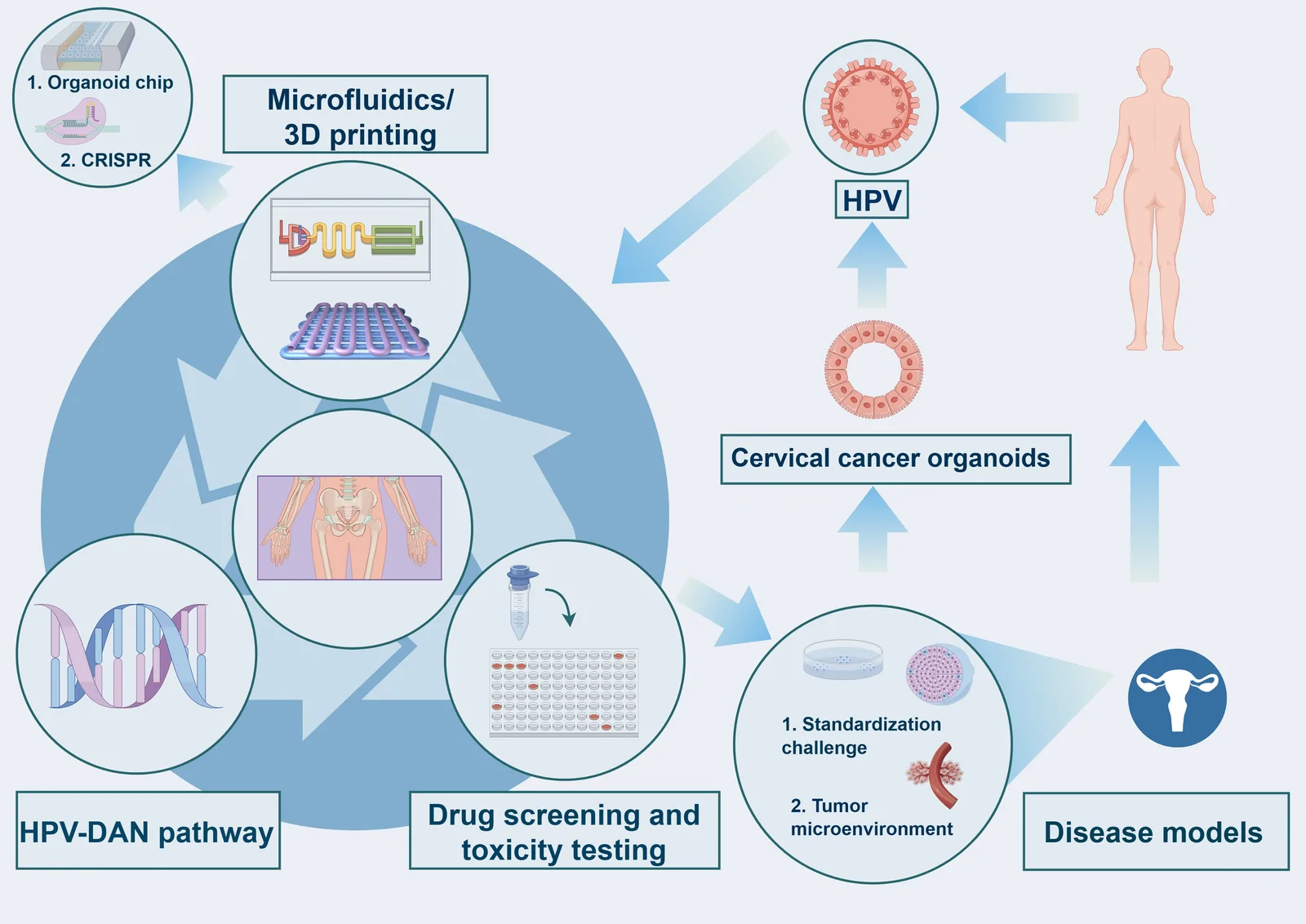
Schematic representation of cervical cancer organoid development and applications.

Based strictly on the gaps and emerging hotspots identified in our bibliometric mapping, future research network expansions are projected to concentrate on: 1) Standardizing high-throughput screening platforms to solidify the “drug discovery” node; 2) Combining CRISPR with organoids to mechanically dissect findings from the “HPV virology” cluster; 3) Validating organoids for radiotherapy prediction; 4) Maturing the integration of AI and microfluidic “organ-on-a-chip” systems to simulate dynamic TME interactions, thereby bridging the highly cited fundamental virology research definitively with clinical immuno-oncology (**Figure 11**).

### 4.2 Strengths and constraints

Bibliometric analysis provides a macroscopic perspective for the study of cervical cancer organoids and can systematically reveal the research hotspots, development trends and cooperation networks in this field. Firstly, the method based on bibliometrics can objectively quantify research outputs, identify high-impact authors, institutions and countries, thereby promoting international scientific research cooperation (29). Secondly, keyword co-occurrence analysis and time evolution maps can visually display the evolution of research topics, such as from the optimization of early organoid culture techniques to the simulation of tumor microenvironments and applications in precision medicine in recent years. Third, citation analysis enables the recognition of landmark foundational studies (43), helping researchers rapidly grasp the core progress of this research area. Collectively, these advantages confirm that bibliometrics serves as a powerful tool to guide future research layouts.

Nevertheless, this study has several inherent limitations. Literature retrieval was performed from three mainstream databases: Web of Science, Scopus, and PubMed. These databases contain high-quality peer-reviewed journals with reliable data authority, powerful citation analysis functions, and mature subject classification systems for accurate literature screening (44–46). However, some open-access and interdisciplinary studies may not be fully covered, which could affect the completeness of the dataset (47). Moreover, we only included English-language publications, which inevitably results in selection and language bias. Research contributions from North America and Europe are relatively overrepresented, while regional non-English journals and academic achievements from non-English-speaking countries cannot be fully reflected (48). Such bias is a common shortcoming of bibliometric studies based solely on English databases. In addition, bibliometric analysis mainly relies on quantitative statistics, which cannot thoroughly evaluate the scientific quality or clinical translational value of individual studies. High-frequency keywords often reflect research hotspots rather than substantive research breakthroughs. Furthermore, emerging research trends, such as the combination of organoid technology and artificial intelligence, may be underrepresented due to publication lag (49). To compensate for these deficiencies, future studies may integrate multi-database and multilingual literature resources, and combine quantitative bibliometric results with qualitative content analysis or expert interviews to achieve a more comprehensive and objective understanding of this field.

Beyond the above biases, there are five additional inherent limitations of this bibliometric analysis that require explicit discussion:

a. Limitations of citation-based metrics
Citation frequency and burst strength only mirror the academic attention received by publications, rather than intrinsic scientific quality. Landmark negative experimental findings or high-value exploratory pilot studies with low citation counts cannot be fully captured via citation-based quantitative indicators.
b. Exclusion of grey literature and preprints
We excluded conference abstracts, non-peer-reviewed preprints, institutional research reports and non-indexed dissertations during screening. This filtering rule may omit preliminary cutting-edge data on cervical cancer organoid immunotherapy that have not yet been formally published in peer-reviewed journals.
c. Citation time-lag bias
Studies published in 2025–2026 have a limited citation accumulation window. Their citation burst strength and co-citation weights are inevitably underestimated, which may reduce the prominence of the most recently emerging immunotherapy research frontiers in our mapping results.
d. Potential self-citation bias
Author- and institutional self-citations may artificially inflate the citation counts of core research teams, leading to overestimation of their centrality and influence in global collaboration networks. We did not remove self-citations before analysis, which constitutes an unavoidable limitation of this work.
e. Inherent constraints of bibliometric clustering algorithms
Clustering outputs from VOSviewer and CiteSpace depend on preset co-occurrence and co-citation similarity thresholds. Our minimum keyword occurrence threshold of 5 may filter weakly connected emerging terms related to immunocompetent organoids. Moreover, the Modularity Q index only evaluates statistical clustering coherence and cannot provide biological validation of thematic grouping rationality.

### 4.3 Impact of global resource disparities on research development

Our bibliometric mapping reveals a conspicuous geographical imbalance in the production of high-impact science. As clearly visualized in our geographical output and collaboration networks (**Figures 3A, C**), the intellectual core of this field is heavily concentrated in North America, Europe, and East Asia (principally China and Japan). Nodes representing these high-income regions are massive and centrally positioned with dense collaborative links. Conversely, nodes representing low- and middle-income countries (LMICs) - particularly regions in Sub-Saharan Africa and South Asia - are notably absent from the core map or relegated to the extreme periphery with minimal link strength. Over 90% of the top 25 highly cited publications originate from European and American institutions (**Figure 7**). This global research imbalance is consistent with the general academic disparity pattern observed in broader gynecologic oncology research.

Notably, this geographical research imbalance mismatches the real clinical demand for cervical cancer research in LMICs. The concentration of fundamental research networks in high-income countries (HICs), as reflected by the dense transatlantic nodes in **Figure 3D**, can be attributed to several interconnected barriers: (1) Financial and infrastructural constraints: Establishing robust organoid biobanks requires sustained investment in sophisticated laboratory infrastructure, advanced equipment, and costly reagents (e.g., Matrigel, recombinant growth factors), which are often scarce in under-resourced settings. (2) Limited access to annotated clinical samples: While LMICs bear the highest disease prevalence, they frequently lack the integrated clinical-research pipelines necessary for the ethical and standardized collection, processing, and storage of patient-derived tissues, which are the foundational material for organoid generation. (3) A gap in technical expertise and training: The specialized skills required for advanced 3D culture techniques are often concentrated within well-funded institutions in HICs, creating a knowledge-access barrier.

This imbalance carries significant implications. It risks generating a scientific knowledge base that does not fully represent the biological heterogeneity-driven by genetic, environmental, and viral strain variations-of cervical cancers in diverse global populations. Consequently, therapeutic strategies and drug sensitivity profiles derived primarily from models based on Western populations may have limited applicability in other contexts, potentially exacerbating global health inequities and hindering the development of effective, localized precision medicine solutions.

Addressing this disparity is paramount for the equitable advancement of the field. Future strategies must prioritize: (1) Fostering equitable international partnerships that move beyond mere sample procurement to encompass co-leadership, co-development, and genuine capacity building, including technology transfer and training programs. (2) Establishing targeted funding initiatives by global health organizations and funders specifically designed to support the establishment of organoid research cores and biobanks in LMICs. (3) Innovating low-cost, accessible protocols, such as developing synthetic hydrogel alternatives to expensive biological matrices and optimizing cost-effective culture media, to lower the economic barriers to entry.

By actively working to mitigate these resource disparities, the scientific community can ensure that the transformative promise of cervical cancer organoid research is realized globally, ultimately benefiting all patients, irrespective of their geographic or socioeconomic circumstances.

## 5 Conclusions

This bibliometric analysis maps the rapid growth of cervical cancer organoid research since 2018, led by contributions from the USA, China, Europe, and Japan. The field has evolved from foundational work on HPV virology and 3D culture towards current focus on tumor microenvironment modeling, high-throughput drug screening, and immunotherapy. While interdisciplinary publication networks and recent high-impact studies signal strong translational momentum, clinical application remains at an early stage. Key challenges in model standardization and subtype-specific culture success must be addressed through advanced co-culture systems, AI-integrated data analysis, and equitable global collaboration to fully realize the potential of organoids in precision oncology.

## Data Availability

The raw data supporting the conclusions of this article will be made available by the authors, without undue reservation.
